# Supplementary material for: Vibrational Spectroscopy Coupled to a Multivariate Analysis Tiered Approach for Argentinean Honey Provenance Confirmation
Source: Foods. 2020 Oct 13;9(10):1450. doi: 10.3390/foods9101450 (PMC7601766; doi:10.3390/foods9101450)
Supplement: Supplementary file 1 [file foods-09-01450-s001.pdf]

foods

Supplementary Information for:

Vibrational Spectroscopy Coupled to a Multivariate Analysis Tiered Approach for Argentinean Honey Provenance Confirmation

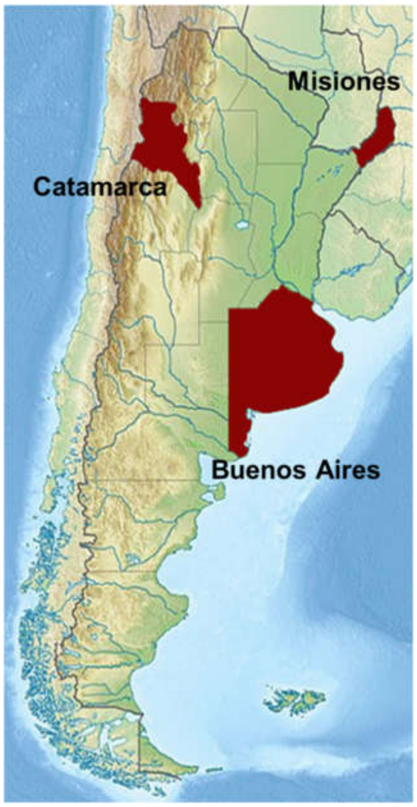

Figure S1. Geographical map of the three Argentinian provinces included in the study.

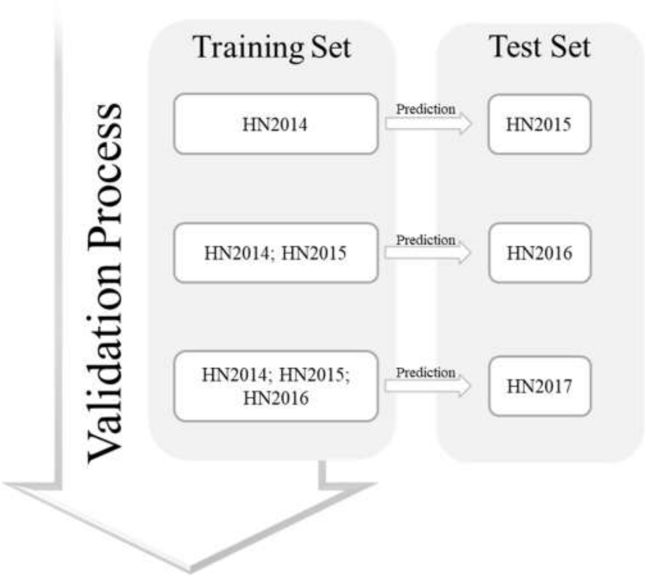

Figure S2. Scheme of the adopted validation strategy.

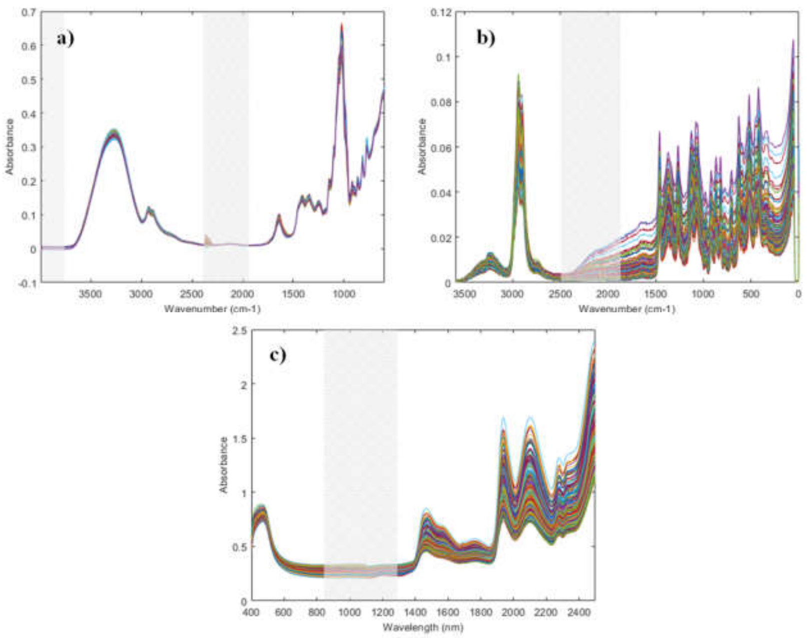

Figure S3. Recorded raw spectra of FT-MIR (**a**), FT-Raman (**b**) and NIR (**c**). Grey bands show spectral regions excluded from the data analysis.

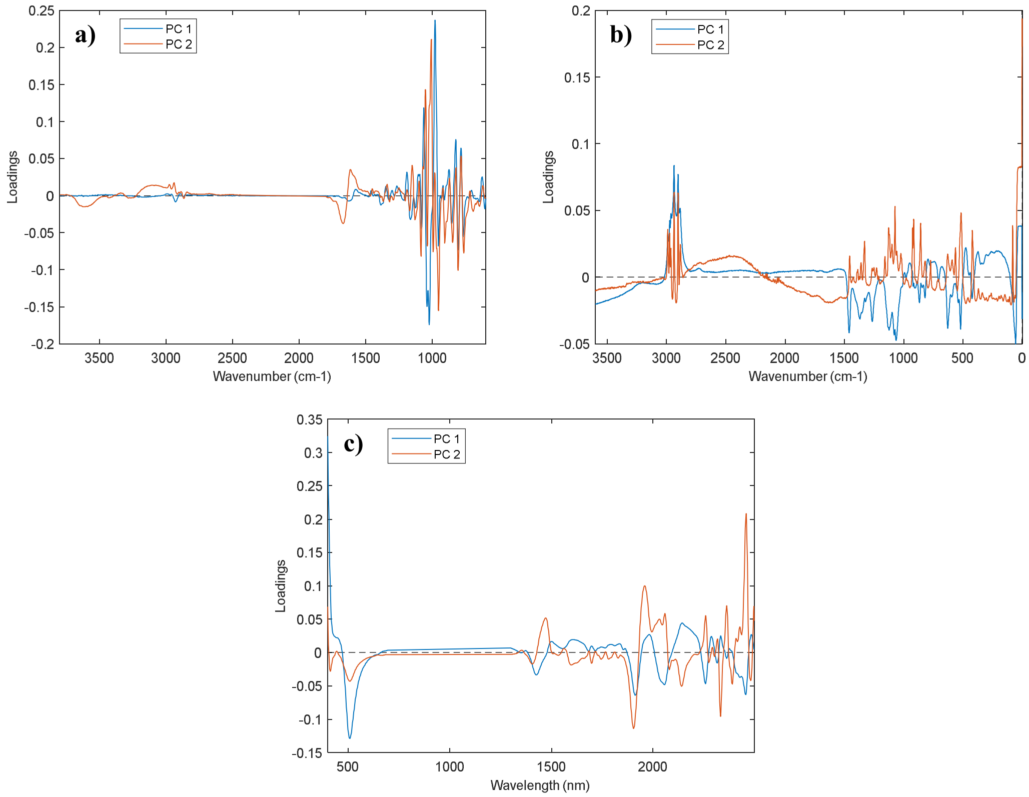

Figure S4. Line plots of PC1 and PC2 loadings obtained from FT-MIR (a), FT-Raman (b) and NIR (c) data.

Band assignment

Investigation of the main peaks/bands relevant for the samples segregation was carried out with the aid of Variable Importance in Projection (VIP) extracted from a PLS model. VIP scores summarize the influence that the original descriptors have had on the PLS model generation. In addition, these scores are scaled in a way that variables exhibiting VIP values greater than 1 can be considered statistically-relevant in a given model. In order to obtain such importance estimates, PLS2-DA models were generated considering all the geographical regions at once (i.e. BA vs Cat vs Mis) on the data coming from the different instrumental techniques. A 5-fold cross validation (venetian blinds) was adopted. Illustration of the VIP scores for the three classification models are reported in **S. Fig 5**.

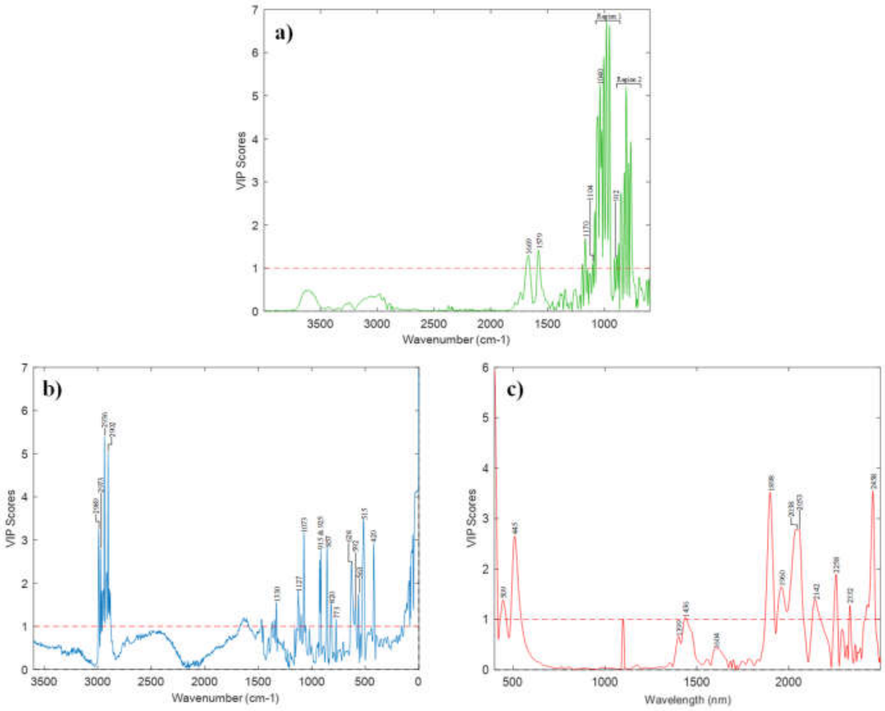

Figure S5. Line plots of the VIP scores extracted from the PLS2-DA (i.e. BA vs Cat vs Mis) model generated on FT-MIR (a), FT-Raman (b) and NIR (c) data. The red dashed line represents the threshold value for significant contribution (VIP=1). Wavenumber/wavelength corresponding to the maximum band absorbance are highlighted.

Concerning the FT-MIR data, as can be seen, Region 1 contains the highest VIP scores, with peaks at 956, 983 and 1006, 1024, 1040, 1061, 1086 and 1104 cm^{-1}. According to the literature, Region 1 (1120–940 cm^{-1}) corresponds to the C–O and C–C stretching of the three major carbohydrates of honey: fructose, glucose and sucrose [1]. Indeed, the peak at 1040 cm^{-1} has been assigned to the C–O stretch in the C–OH group in the carbohydrate structure [2,3]. Furthermore, the small peak at 1104 cm^{-1} corresponds to the C–O stretching band of the C–O–C linkage (the C–O–C is present in sucrose as a glycoside bond) [2]. High VIP values can be also observed in Region 2 (920–750 cm^{-1}) with peaks at 767, 784, 806, 824, 854 872, 889 and 912 cm^{-1}. This is the anomeric region, characteristic of the saccharide configuration [1,2]. In particular, the peak at 912 cm^{-1} is due to the C–H bending of the carbohydrate. No statistically relevant bands were highlighted in the 1540–1175 cm^{-1} range, which has been assigned by Gok and co-workers to the O–H stretching/bending, C=O stretching of ketones and the C–O & C–H stretching of carbohydrates [4].

With regard to FT-Raman data, the two peaks at 2936 and 2902 cm^{-1} are related to the asymmetric stretching of CH_2 and C-H stretching vibrations, respectively. In addition, several peaks characteristic

of different chemical groups can be observed in the fingerprint region (200-1500 cm^{-1}). Most of these signals are consistent with the literature and thorough band assignment can be found in our previous work [5].

Finally, VIP extracted from NIR data highlighted two statistically-relevant bands, 1400-1490 nm and 1870-1930 nm, both corresponding to O–H, C–H, and C–H_2 deformations [6]. Besides, the spectral range between 2000 and 2100 nm corresponds to the C–H combinations [6,7], the minor peak at 2332 nm has been related to the C–H bonds [8] and the 2430-2490 nm band is characteristic of the O–H, N–H, and C–H deformation [6]. Furthermore, signals at 1470 and 1960 nm have been associated with the O-H stretch (first and second overtone, respectively) [9].

Table S1. Sample set overview.

Year	Buenos Aires	Catamarca	Misiones
2014	78	25	31
2015	93	11	28
2016	107	15	25
2017	45	17	28

Table S2. PLS-DA prediction results expressed as correct classification rates (FT-Raman data).

Predicted harvest	Correct classification rate (%)		
	**BA vs Cat**	BA. vs Mis	Cat vs Mis
2015	80.7	95.8	79.4
2016	81.9	96.2	80.0
2017	82.3	97.2	84.4

Table 3. PLS-DA prediction results expressed as correct classification rates (NIR data).

Predicted harvest	Correct classification rate (%)		
	**BA vs Cat**	BA. vs Mis	Cat vs Mis
2015	70.1	96.6	76.5
2016	80.3	87.1	65.0
2017	88.7	94.5	82.2

References

1. Subari, N.; Mohamad Saleh, J.; Md Shakaff, A.; Zakaria, A. A Hybrid Sensing Approach for Pure and Adulterated Honey Classification. *Sensors* **2012**, *12* (10), 14022–14040. https://doi.org/10.3390/s121014022.
2. Gallardo-Velázquez, T.; Osorio-Revilla, G.; Loa, M. Z. de; Rivera-Espinoza, Y. Application of FTIR-HATR Spectroscopy and Multivariate Analysis to the Quantification of Adulterants in Mexican Honeys. *Food Res. Int.* **2009**, *42* (3), 313–318. https://doi.org/10.1016/j.foodres.2008.11.010.
3. Tewari, J. C.; And, †; Irudayaraj, J. M. K. Floral Classification of Honey Using Mid-Infrared Spectroscopy and Surface Acoustic Wave Based z-Nose Sensor. **2005**. https://doi.org/10.1021/jf050139z.
4. Gok, S.; Severcan, M.; Goormaghtigh, E.; Kandemir, I.; Severcan, F. Differentiation of Anatolian Honey Samples from Different Botanical Origins by ATR-FTIR Spectroscopy Using Multivariate Analysis. *Food Chem.* **2015**, *170*, 234–240. https://doi.org/10.1016/j.foodchem.2014.08.040.
5. Fernándz Pierna, J. A.; Abbas, O.; Dardenne, P.; Baeten, V. Discrimination of Corsican Honey by FT-Raman Spectroscopy and Chemometrics. *Biotechnol. Agron. Soc. Environ.* **2011**, *15* (1), 75–84.
6. Bisutti, V.; Merlanti, R.; Serva, L.; Lucatello, L.; Mirisola, M.; Balzan, S.; Tenti, S.; Fontana, F.; Trevisan, G.; Montanucci, L.; Contiero, B.; Segato, S.; Capolongo, F. Multivariate and Machine Learning Approaches for Honey Botanical Origin Authentication Using near Infrared Spectroscopy. *J. Near Infrared Spectrosc.* **2019**, *27* (1), 65–74. https://doi.org/10.1177/0967033518824765.
7. Woodcock, T.; Downey, G.; Kelly, J. D.; O'Donnell, C. Geographical Classification of Honey Samples by Near-Infrared Spectroscopy: A Feasibility Study. *J. Agric. Food Chem.* **2007**, *55* (22), 9128–9134. https://doi.org/10.1021/jf072010q.
8. Latorre, C. H.; Crecente, R. M. P.; Martín, S. G.; García, J. B. A Fast Chemometric Procedure Based on NIR Data for Authentication of Honey with Protected Geographical Indication. *Food Chem.* **2013**, *141* (4), 3559–3565. https://doi.org/10.1016/j.foodchem.2013.06.022.
9. Corbella, E.; Cozzolino, D. The Use of Visible and near Infrared Spectroscopy to Classify the Floral Origin of Honey Samples Produced in Uruguay. *J. Near Infrared Spectrosc.* **2005**, *13* (2), 63–68. https://doi.org/10.1255/jnirs.458.
